# Phenotypic characters of rice landraces reveal independent lineages of short-grain aromatic *indica* rice

**DOI:** 10.1093/aobpla/plt032

**Published:** 2013-08-01

**Authors:** Avik Ray, Debal Deb, Rajasri Ray, Balaji Chattopadhayay

**Affiliations:** 1 NCBS-TIFR, Ecology and Evolution, Bellary Road, Bangalore, Karnataka 560065, India; 2 Centre for Interdisciplinary Studies, 9 Old Calcutta Road, Barrackpore, Kolkata 700123, India; 3 Center for Ecological Sciences, Indian Institute of Science, Bangalore, Karnataka 560012, India; 4 A Step Forward, 1601 Pearapur Road, Sheoraphuli, West Bengal 712223, India; 5 Present address: Ashoka Trust for Research in Ecology and the Environment (ATREE), Royal Enclave, Sriramapura, Jakkur Post, Bangalore 560064, India

**Keywords:** Aromatic, Basmati, domestication, grain length, landraces, phenotypic diversity, rice

## Abstract

Rice landraces are lineages developed by farmers through artificial selection during the long-term domestication process. Despite huge potential for crop improvement, they are largely understudied in India. Here, we analyse a suite of phenotypic characters from large numbers of Indian landraces comprised of both aromatic and non-aromatic varieties. Our primary aim was to investigate the major determinants of diversity, the strength of segregation among aromatic and non-aromatic landraces as well as that within aromatic landraces. Using principal component analysis, we found that grain length, width and weight, panicle weight and leaf length have the most substantial contribution. Discriminant analysis can effectively distinguish the majority of aromatic from non-aromatic landraces. More interestingly, within aromatic landraces long-grain traditional Basmati and short-grain non-Basmati aromatics remain morphologically well differentiated. The present research emphasizes the general patterns of phenotypic diversity and finds out the most important characters. It also confirms the existence of very unique short-grain aromatic landraces, perhaps carrying signatures of independent origin of an additional aroma quantitative trait locus in the *indica* group, unlike introgression of specific alleles of the *BADH2* gene from the *japonica* group as in Basmati. We presume that this parallel origin and evolution of aroma in short-grain *indica* landraces are linked to the long history of rice domestication that involved inheritance of several traits from *Oryza nivara*, in addition to *O. rufipogon*. We conclude with a note that the insights from the phenotypic analysis essentially comprise the first part, which will likely be validated with subsequent molecular analysis.

## Abstract (precis)

Crop domestication is a remarkable example of evolution of wildly growing plants into cultivable forms through human selection. Following the domestication of rice almost 10,000 years ago, ancient farmers selected many rice lineages for diverse agronomic and cultural traits, like grain size, shape and colour; awn length; pest resistance; and aroma etc. In this study, examining phenotypic traits of a large collection of Indian rice landraces (all accessed from Vrihi, rice seed bank, www.cintdis.org/vrihi) we characterize the huge phenotypic diversity, and find that a few grain, panicle and leaf traits are major drivers of this diversity. We also demonstrate the existence of short grain aromatic landraces perhaps with independently evolved aroma trait; unlike introgression from *japonica* into *indica* group, as evidenced in Basmati-type long grain varieties. The independent origin of aroma in *indica* rice is fascinating as it explores lesser known aspects of *indica* rice domestication and diversification.

## Introduction

Crop domestication is a complex process mediated by a series of prolific phenotypic changes to modify a wild species so that it is amenable to cultivation, harvest as well as consumption. Continuous selection of desirable phenotypic traits from the wild species *Oryza rufipogon* gradually gave rise to the domesticated *O. sativa* that now feeds billions of people globally (Khush 1997; Kovach *et al*. 2007). Based on several morphological and genetic markers, *O. sativa* is broadly divided into two varietal groups, namely *japonica* and *indica*, and these groups are further subdivided into five distinct subpopulations (Garris *et al*. 2005). Apart from that, considerable morphological, ecological and physiological variations exist within each varietal subpopulation owing to selection for adaptations to different agro-climatic conditions (Khush 1997).

Several independent domestication events might have occurred to establish cultivated rice in China, India and Southeast Asia (Konishi *et al*. 2006). Rice landraces are the groups of lineages that originated and evolved in the field over millennia through selective breeding by generations of farmers, who chose random mutants and gene combinations in domesticated rice, for better yield, grain size and other agronomic or cultural values (Deb 2005). Selective breeding, random mutation as well as frequent hybridization between the landraces and wild relatives over a long time ensured the accumulation of a high phenotypic as well as genetic diversity (Fukuoka *et al*. 2006; Sanni *et al*. 2008). Retention of immense genetic diversity is not only significant in terms of evolutionary potential to withstand diverse selection regimes, but also has important implications in rice breeding to furnish new genes for crop improvement, e.g. abiotic stress tolerance, or pest- or disease-resistance genes (Frankel and Soule 1981). However, beginning from the 1960s, a large number of these landraces have been replaced with modern varieties introduced over the past four decades (Heal *et al*. 2004; Deb 2009).

*Indica* landraces can be identified based on several morphological features, e.g. plant height, leaf length and width, grain weight, size and colour, presence/absence of awn and aroma, etc. (Deb 2005). In most cases, the selection of morphological traits is based on certain inherent qualities, e.g. adaptation to marginal environmental conditions (e.g. flood, drought and soil salinity) or specific agronomic and cultural traits (early maturity, aroma, medicinal properties and high grain yield), etc. India being a centre of rice diversity, it can be imagined that these landraces have imbibed enormous variation from recurrent mutations and from their wild ancestors through ages of agricultural practices; however, owing to the predominant use of modern high-yielding varieties (HYV), a massive proportion of the indigenous rice genetic diversity has already disappeared from farmers' fields (Chang 1984). Therefore, assessment, documentation, analysis and conservation of the extant genetic diversity are essential prerequisites to mine useful genes for the development of the new, adaptive cultivars (Chang 1984). Also importantly, these landraces represent intermediate forms that are genetically well differentiated from wild relatives but still not exploited in modern breeding experiments for cultivar development. So, presumably they possess ancient signals of domestication, e.g. specific allelic combinations that may be extremely valuable for gaining insights into early rice domestication events.

In this paper, we evaluate a suite of 29 phenotypic characters from 414 rice landraces (both aromatic and non-aromatic) to investigate the major determinants of phenotypic diversity. We further investigated the morphological distinction between aromatic and non-aromatic landraces as well as that within aromatic landraces.

## Methods

### Seed collection and conservation

Folk rice varieties were collected from farmers' fields of various Indian states (Deb 2005). Seeds of these farmer landraces comprise the accessions of *Vrihi* (www.cintdis.org/vrihi), the country's largest non-governmental seed bank conserving 920 folk rice varieties. All these varieties have been grown every year on Vrihi's conservation farms, located in the district of Bankura (West Bengal, India) and Rayagada (Odisha, India), over the past 17 years (www.cintdis.org/basudha). Genetic purity of each landrace is maintained by periodic rouging of ‘off types’, in addition to obviating chances of cross-pollination between varieties grown on neighbouring farm plots by employing the flowering asynchrony method (Deb 2006). From the total accession of 920 farmer landraces, 414 were selected for this study.

The germplasms of most of the rice landraces are publically available at Vrihi (www.cintdis.org/vrihi) upon request for scientific study except for commercial research affiliated to corporate sector.

### Experiments and measurements of agronomic traits

Each of the rice landraces was grown every year on a 2 m × 2 m plot, from which 10 hills were sampled for characterization. Morphological and agronomic traits of each landrace were recorded every year. For this study, 29 phenotypic characters from the selected 414 landraces at different growth stages were measured following the International Rice Research Institute (IRRI 1980) and International Network for Genetic Evaluation of Rice (INGER 1996) guidelines. Parts of this documentation have been published elsewhere (Deb 2005). The rice characters selected for this study and their units of measurements are listed with abbreviations **[see** **Supporting Information —Table S1]**.

### Data analysis

We examined the relative locations of different landraces in the morpho-space by means of principal coordinate analysis (PCoA) using all the 29 variables in PAST (Hammer *et al*. 2001). Next, we generated a frequency distribution plot for all continuous variables among the 29 characters, followed by a principal component analysis (PCA) with continuous variables, to find out the overall pattern of phenotypic diversity as well as variable contributions to diversity. We initially performed PCA with 14 continuous variables with their log-transformed values and finally scaled down to eight variables based on internal tests (i.e. anti-image correlation matrix, KMO–Bartlett's test of sphericity and communality) for PCA suitability in SPSS ver. 19.0 (SPSS, Inc., Chicago, IL, USA). In the next step, we divided all the landraces into two *a priori* groups (aromatic and non-aromatic) based on the broad trend observed in the PCA and made an attempt to find the best variable(s) that can discriminate the groups. Subsequent analyses involved forward stepwise discriminant function analysis (DFA) with eight characters (100-grain weight [SW], grain length [GL], grain width [GW], decorticated grain width [DW], decorticated grain length [DL], panicle density [PD], leaf length [LL] and plant height [HT]) to understand the combination of variables which can best explain the grouping in STATISTICA ver. 10 (StatSoft, Inc., Tulsa, OK, USA). We performed DFA considering F to enter as 0.01, F to remove at 0.0 and minimum tolerance at the default value of 0.01. We also obtained a classification function, which can possibly explain the grouping. This function can be used for further cross-validation. Following this, we checked whether group means are significantly different by Welch two-sample *t*-test in R 2.15.2 (R Development Core Team 2011).

Finally, we re-examined the morphological distinction within the aromatic group between traditional Basmati and non-Basmati aromatic landraces through 3-D scatterplot and neighbour-joining cluster analysis in PAST (Hammer *et al*. 2001). In doing so, we combined the published dataset (Takano-Kai *et al*. 2009) consisting of grain length, width and weight of several global accessions of *O. sativa* with our data. The rationale behind such an analysis is to check whether the distinction between two different aromatic groups, i.e. Basmati and non-Basmati, persists when a major fraction of global variation of grains is added to our analysis.

## Results

### Phenotypic variation

#### Univariate statistics

The landraces have a wide diversity of morphological characters. Specifically, some characters like SW, GL, GW, DL, DW, PD, LL, %ST, PW and HT show varying degrees of polymorphism, whereas F, AL, LA, FA, TH, AC, P and LP are almost monomorphic [**see Supporting Information—Fig. S1**].

#### Multivariate statistics

**Figure 1.**
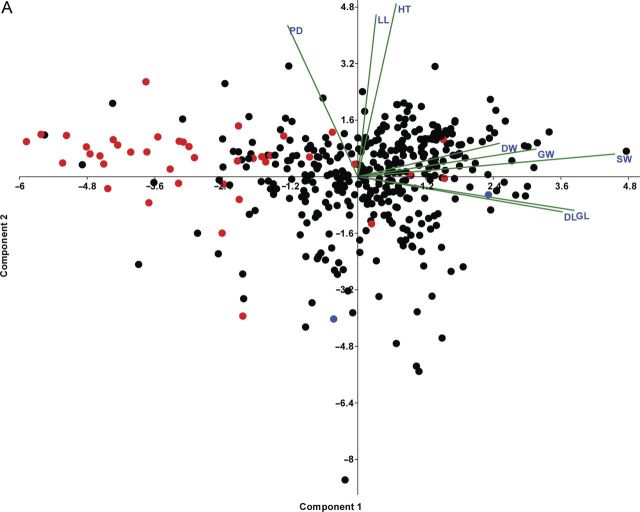

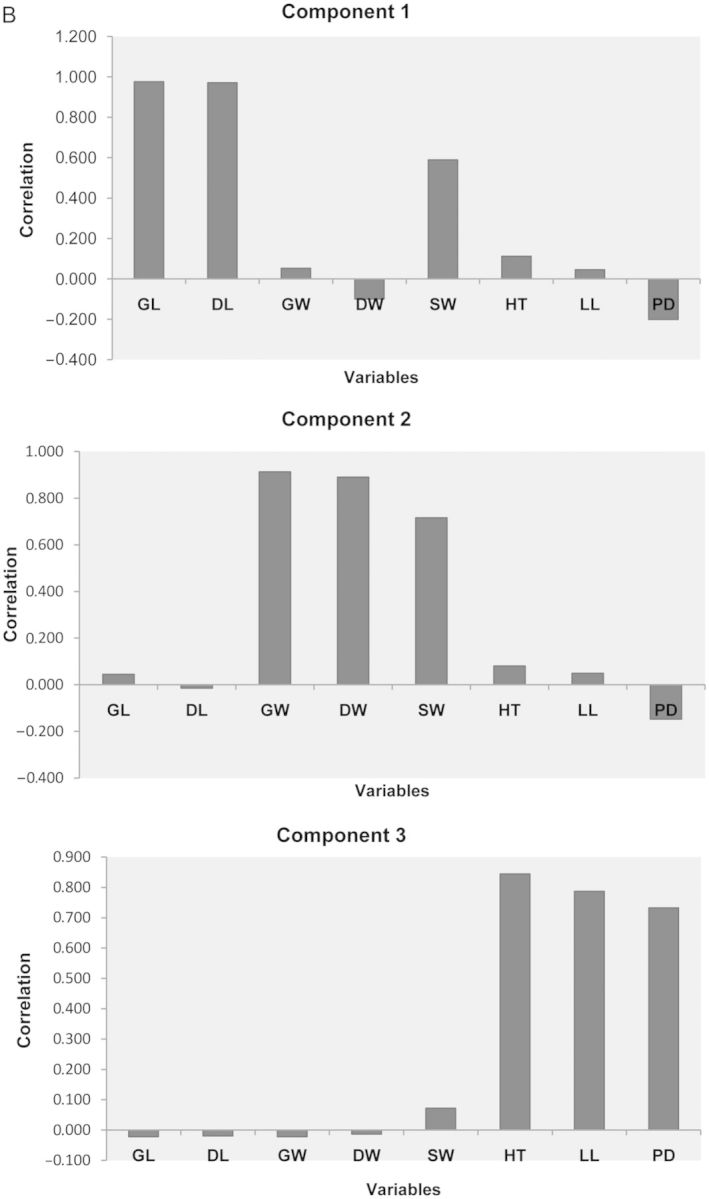
PCA with continuous characters of 414 landraces, (A) showing separation among landraces and within aromatic landraces. The non-Basmati aromatic landraces, non-aromatic landraces and Basmati along with Dehradun gandheswari are shown as red, black and blue circles, respectively; (B) showing effective loading into first, second and third principal components.

Principal coordinate analysis with all 29 variables (continuous and categorical) depicted effective segregation of a majority of aromatic and non-aromatic landraces [**see Supporting Information—Fig. S2**]. In PCA, the first, second and third component explained 29.11, 27.36 and 23.04 % (totalling 79.52 %) of the variance, respectively. Grain length and DL are highly correlated with component 1 (*r*^2^ = 0.97 for both), GW, DW and SW are correlated with component 2 (*r*^2^ = 0.91, 0.89 and 0.71, respectively) and HT, LL and PD are related to component 3 (*r*^2^ = 0.84, 0.78 and 0.73, respectively) (Fig. 1A and B). Based on the loading and biplot, it appeared that a few morphological characters are the major determinants of phenotypic diversity, among which grain length, weight and width, leaf length, panicle density and plant height play a pivotal role. The rest of the characters seem to have a very minimal contribution to variability.

We have found some general patterns created by the principal components. Heavier grains are larger in size (e.g. Patnai, Gochari Patnai, Sabita-Patnai, Ganga-Sal, Langal-Muda, Sada Patnai, Dangri Patnai, Keshab-Sal, Patnai-23, Bakul phool, Gitanjali, Sada-Jhinga-Sal, Basmati etc.). A majority of aromatic landraces, except Basmati, have mostly small and lighter grains, as shown by Tulsibhog, Lilabati, Dar sal, Tulsi mukul, Karpurtal, Kanakchur, etc. Apart from these there are other non-aromatics like Begana manjia, Darka sal, Zeeni, Pakri, Shishaphal, Jirkudi, Rani kajal and Ranjit with shorter and lighter grains. Panicle density, leaf length and plant height are also important discriminating factors; tall plants tend to have long leaves and denser panicles, e.g. Bochi gondri, Begana manjia, Kajal dekhi, Ajirman, Agniban, Jal kamini, Sholeh, etc., whereas shorter plants seem to have smaller leaves and panicles, e.g. Gitanjali, Mugi mansara, Lal sita, Sundar mukhi, Gour nitai, Sonam, etc.

### Difference between aromatic and non-aromatic landraces

The first two components of the PCA scatter plot have effectively separated most of the aromatic landraces from the non-aromatic group; most of the aromatic landraces have smaller and lighter grains. However, exceptionally long-grain Basmati and Dehradun gandheswari do not fall into this group and are located far apart from the major cluster of aromatics in morpho-space, and a few lie in between the extremes (e.g. Radhashree and Parmaisal).

Nevertheless, DFA with SW, GL, HT and GW has effectively validated the *a priori* grouping of aromatic and non-aromatic landraces. A model consisting of four variables, out of eight selected from PCA (Table 1), was found to best explain the grouping. We did not observe absolute discrimination between the two groups, and we were able to score 88.4 % correct classification. The discriminant function is statistically significant with moderate canonical correlation *R* = 0.541 and group separation (eigenvalue, 0.415542; canonical *R,* 0.541809; Wilks' lambda, 0.706443; cumulative probability, 100 %; *P* < 0.01). It appears that SW can best explain the grouping between aromatic and non-aromatic groups. In the model, it has the lowest partial lambda, the highest standardized coefficient, and F-remove values. By itself, SW can classify 82 % of the data correctly to their groups. The contribution of GL is also quite significant in the model, and it is more evident from its higher tolerance as SW and moderate values of other parameters. Furthermore, Basmati and Dehradun gandeshwari were misclassified into the non-aromatic group. The classification function can correctly classify 77 % of the aromatic and 89.7 % of the non-aromatic varieties. This grouping can be further used for cross-validation and assignment of unknown samples. We then performed a Welch two-sample *t*-test, which showed that the group means are significantly different (*t* = 8.2154, df = 47.765, *P* < 10^–9^). The non-aromatic landraces have heavier grains (mean GW ∼ 1.24 g) than the aromatic landraces in general (mean GW ∼ 0.98 g). We also performed a Hotelling *T*^2^ test including both SW and GL and found that both have significantly different group means (data not shown).

**Table 1.** Summary of discriminant analysis with 414 aromatic and non-aromatic landraces. Number of variables in model, 4; Wilks' lambda, 0.706 approximately, *F*(4,409) = 42.489, *P* < 0.0000.

Characters	Wilks' lambda	Partial lambda	F-remove (1,554)	*P*-value	Tolerance	1-Toler. (*R*^2^)	Standardized coefficient
100-Seed weight (SW)	0.746	0.945	23.402	0.000002	0.357	0.642	0.717
Grain length (GL)	0.742	0.951	20.871	0.000007	0.625	0.374	0.514
Plant height (HT)	0.711	0.992	2.924	0.088	0.953	0.046	−0.159
Grain width (GW)	0.707	0.998	0.663	0.415	0.462	0.537	−0.109

### Differences in grain dimensions among aromatic landraces

**Figure 2.**
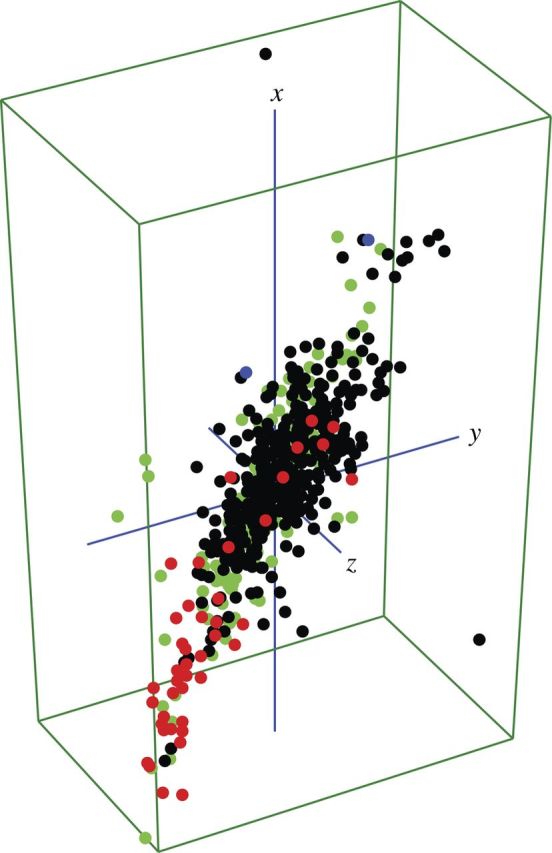
Three-dimensional scatter plot with grain weight, length and width of global accessions of *O. sativa* as well as 414 landraces showing separation between Basmati and non-Basmati aromatic landraces. The non-Basmati aromatic landraces, non-aromatic landraces and Basmati along with Dehradun gandheswari, global *O. sativa* accessions are shown as red, black, blue and green circles, respectively.

Examination of additional landraces from different globally diverse *O. sativa* accessions, in combination with our own data set, revealed a conspicuous intra-aromatic separation in the 3D scatter plot and cluster diagram on the basis of major variations in grain length, width and weight (Fig. 2) [**see Supporting Information—Fig. S3**]. Traditional Basmati-types and non-Basmati aromatic landraces from both the samples from global *O. sativa* accessions and our own landraces were clearly separated into two distinct groups.

## Discussion

Rice landraces have evolved from their wild progenitor mostly by anthropogenic and natural selection (Zong *et al*. 2007) and nevertheless retain huge genetic diversity (Frankel and Soule 1981; Nguyen 2002). These indigenous farmer landraces can tolerate a wide range of environmental stress, resulting in highly stable and an intermediate yield in low-input agricultural systems (Huang *et al*. 2010), and can substantially enrich the gene pool of advanced cultivars (Chang 1984; Fukuoka *et al.* 2006).

India is one of the major centres of rice diversity, but little initiative has been taken to conserve and assess the landrace diversity; rather, an aggressive campaign to promote a handful of modern HYV and hybrids has caused a rapid erosion of the indigenous rice genetic diversity, resulting in the disappearance of thousands of landraces from farm fields (Jackson 1994, 1995; Deb 2005, 2009; Yamasaki *et al*. 2005). Elaborate morphological description and analyses are rare for indigenous rice landraces. So far the only available documentation of a wide range of morphological and culturally important characters of *indica* rice landraces is Deb (2005); however, this work involved no further analysis of the characters. So far, all studies in rice genetic diversity have mostly dealt with small sets of rice landraces, e.g. aromatic rice (Ray Choudhury *et al*. 2001), lowland rice of Assam (Bhuyan *et al*. 2007), stress tolerant varieties (Reddy *et al.* 2009) and tall landraces (Neeraja *et al*. 2005). The present work is a first attempt to investigate the general pattern of phenotypic variation of a set of traits among a significantly large number of rare landraces (some of which are not accessed in international and national gene banks).

The current study validates the effective segregation of aromatic and non-aromatic landraces, based on grain characters chosen from multivariate analyses. It further argues for an independent origin and evolution of the aromatic lineages in *indica* and traces the history of rice domestication, finer details of which can be further elucidated by molecular genetics analyses.

Our analysis indicates that the quantitative grain characteristics (grain length, grain width, grain weight, decorticated grain length and decorticated grain width) along with panicle density, leaf length and plant height predominantly contribute to the observed range of phenotypic diversity. This finding is in agreement with earlier work, e.g. Sanni *et al*. (2008) showed prominent contributions of grain length, grain weight, panicle length, leaf width and tillering ability to the phenotypic diversity in Côte-d'Ivore landraces. The phenotypic variability of African traditional rice is primarily driven by panicle length, grain weight, grain length, grain width, flag leaf width, tiller numbers and days to heading (Meizan 1985). Li *et al*. (2010) demonstrated grain weight as one of the most important characters enhancing variability. Our study also shows leaf length and plant height have a significant contribution to variability in rice landraces, a finding not reported in previous studies.

The prominent contribution of grain length, grain weight and panicle density to rice phenotypic diversity is perhaps due to the fact that the grain and panicle characters are agronomically the most important traits, which were subjected to strong directional and diversifying selection by farmers over generations (McCouch 2004). However, artificial (directional) selection during the early domestication process would have caused a drastic loss of diversity, which in no way accounts for the high degree of variation observed here. Nevertheless, unlike in other cereals, e.g. maize and wheat, selection in rice has not always operated unidirectionally to achieve a uniform increase in rice grain size. Rather, diverse grain sizes and colours were selected at various local geographic scales for specific regional cultural and utilitarian requirements (Fitzgerald *et al*. 2009). The result is that a wide range of grain length (from 5.7 to 11.3 mm) exists in *indica* landraces while a narrow range prevails in its wild ancestors (e.g. 7.32–9.95 mm) (Takano-Kai *et al*. 2009). Similar selection processes enhanced the cooking quality, amylose content and degree of aroma in a large number of landraces in combination with grain size and colour variations. Divergent local preferences for several morphological traits have preserved a wide range of grain width, colour, plant stature, flag leaf angle and various culinary qualities and have led to conservation of enormous allelic diversity. Therefore, we surmise that while early domestication might have eroded a relatively small amount of this diversity, recurrent mutations and farmers' selections over centuries might also have magnified a certain portion of diversity. This argument is in contrast to the prevailing hypothesis about the severe loss of diversity mediated by artificial selection during the domestication process. It is postulated that artificial selection in crops operated in a two-step fashion: the initial loss is during domestication when wild progenitors gave rise to landraces adapted to wider environmental conditions, and next when modern agricultural activities generated almost homogeneous inbred lines from the landraces through strong selection of agriculturally important traits (Yamasaki *et al*. 2005). We argue that the first step also involved enhancement and preservation of the allelic diversity of numerous morphological traits through various local selections and preservation of novel mutants; it is the second step (homogenization) that caused an enormous decline in crop genetic diversity, which continues to take place with the introduction of modern varieties.

Our analysis of all morphological characters and that of selected quantitative characters contradistinguish most of the aromatic varieties from the non-aromatic landraces in the scatterplot. The segregation of aromatic landraces on morpho-space may indicate that some aromatic landraces might have independently evolved into close-knit lineages. This conjecture is further supported by discriminant analysis, which clearly segregates most of the aromatic and non-aromatic landraces into two distinct groups based on grain dimensions, although it misclassified the long-grained Basmati and Dehradun gandheswari into the non-aromatic group. Additionally, the explicit morphological separation of traditional Basmati and non-Basmati aromatic landraces in the PCA scatterplot is another significant outcome of our analysis. This corroborates the geographical distinction of small- and medium-grain non-Basmati aromatics from Basmati-type long-grain aromatic rice (Sharma *et al*. 2000) and is confirmed by our extended analysis of grain characters of additional *O. sativa* accessions from across the world (Fig. 2) [**see Supporting Information—Fig. S3**].

Our interpretation is based entirely on the phenotypic data, which have been routinely used to delimit species, populations and varietal groups in micro- and macro-evolutionary studies (Armbruster *et al.* 2004; Eble 2004). While genetic analysis is necessary to validate our interpretation, phenotypic data may often suffice to correlate species diversification rates and phenotypic divergence (Ricklefs 2004, 2006). Furthermore, the morphological distinction between traditional Basmati and non-Basmati aromatic landraces is robust enough, even on a scale of global variance of grain characters, and is not limited to a certain geographic region.

The prominent difference between long- and short-grain landraces essentially constitutes important evidence of their independent evolution and poses an intriguing question about the origin of aroma. Aroma in Basmati is presumed to have been introgressed from the *japonica* varietal group into the *indica* group (Garris *et al*. 2005; Kovach *et al*. 2007, 2009). In addition, grain length of Basmati-like landraces is also presumed to be introgressed from the *japonica* varietal group (Takano-Kai *et al*. 2009). Therefore, if short-grain non-Basmati aromatic *indica* landraces independently evolved into a divergent lineage, it seems highly likely that an alternative/additional QTL for aroma exists in the *indica* group of rice (Fitzgerald *et al.* 2008). Likewise, the grain elongation QTL may be contributions from *O. rufipogon* and/or *O. nivara*. The discovery of a distinct lineage of annual species in peninsular India derived from *O. nivara* (Sharma *et al.* 2000) and the discovery of rare *O. nivara* alleles shared by Basmati 370 (Sarla *et al.* 2003) lend support to our hypothesis of inheritance of aroma and Basmati-type grain length in *indica* rice from ancestral South Asian species rather than from *O. sativa japonica*.

## Conclusions

Our study highlights the immense phenotypic diversity of conserved landraces. Among the morphological characteristics, grain weight, length and width; decorticated grain length and width; leaf length; panicle density and plant height contributed most to overall variability among *indica* rice landraces. The existence of a wide range of phenotypic traits indicates that landraces were (and continue to be) selected by farmers for diverse cultural and local ecological needs. This has led to preservation, and in some cases, enhancement of the allelic diversity in rice. This inference is in contrast to the widely held view that the domestication process entailed loss of immense genetic diversity.

Our study further emphasizes the distinctive morphological difference between most of the aromatic and non-aromatic landraces, and a clear separation between long-grain Basmati and short-grain non-Basmati aromatic varieties, which indicates the possibile origin of additional aromatic lineages within the *indica* group. Moreover, the two distinct groups of landraces based on grain size appear to contradistinguish the aromatic from the non-aromatic landraces—a feature that is not predictable from the biochemical basis of rice aroma (imparted by 2-acetyl 1-pyrroline; Brahmachary 1996).

These findings seem to pose a challenge to the conjectures that at least some genes or QTLs ‘for’ aroma (Garris *et al.* 2005; Kovach *et al*. 2007, 2009) and genes ‘for’ grain length (Takano-Kai *et al*. 2009) were introgressed from *japonica* into *indica* landraces during early migration of people and their selection of rice lines. The distinctive separation of aromatic short-grain from non-aromatic long-grain landraces may indicate that two separate gene clusters, expressing aroma and short-grain trait are tightly linked, or alternatively, a single gene cluster has pleiotropic effects on aroma and short-grain length, so that long-grain non-Basmati landraces are not aromatic. Of course, our interpretations warrant further molecular analyses, which would essentially follow our initial conclusions to gain fresh insights into the history of rice domestication.

## Sources of Funding

The authors received no financial support for this study.

## Contributions by the Authors

A.R. and D.D. conceived the idea and designed the experiments; D.D. collected morphological data; A.R., R.R. and B.C. analysed the data; A.R. and D.D. wrote the manuscript.

## Conflicts of Interest Statement

None declared.

## Supporting Information

The following files are available in the online version of this article:

**File 1.** Table S1: A list of phenotypic characters and their units of measurements included in this study with their abbreviations used in the text.

**File 2.** Figure S1: Frequency distribution of 29 phenotypic characters measured in 414 landraces. Details of abbreviations and units of measurements are summarized in Table S1.

**File 3.** Figure S2: Principal coordinate analysis with all characters of 414 landraces showing separation among landraces and within aromatic landraces. The non-Basmati aromatic landraces, non-aromatic landraces and Basmati along with Dehradun gandheswari are shown as red, black and blue circles, respectively.

**File 4.** Figure S3: Cluster diagram with grain weight, length and width of global accessions of *O. sativa* as well as 414 landraces showing separation between Basmati and non-Basmati aromatic landraces. The non-Basmati aromatic landraces, non-aromatic landraces, Basmati along with Dehradun gandheswari (Basmati-type), and global *O. sativa* accessions are shown in red, black, blue and green fonts, respectively [trop. japonica = tropical japonica, temp. japonica = temperate japonica].

Additional Information

## References

[PLT032C1] Armbruster WS, Pelabon C, Hansen TF, Mulder CPH, Pigliucci M, Preston K (2004). Floral integration, modularity and accuracy: distinguishing complex adaptations from genetic constraints. Phenotypic integration: studying the ecology and evolution of complex phenotypes.

[PLT032C2] Bhuyan N, Borah BK, Sharma RN (2007). Genetic diversity analysis of traditional lowland rice (Oryza sativa L.) of Assam using RAPD and ISSR markers. Current Science.

[PLT032C3] Brahmachary RL (1996). The expanding world of 2-acetyl-1-pyrroline. Current Science.

[PLT032C4] Chang TT (1984). Conservation of rice genetic resources: luxury or necessity?. Science.

[PLT032C5] Deb D (2005). Seeds of tradition, seeds of future, folk rice varieties of Eastern India.

[PLT032C37] Deb D (2006). Flowering asynchrony can maintain genetic purity in rice landraces. Current Science.

[PLT032C6] Deb D (2009). Valuing folk crop varieties for agroecology and food security. Bioscience Resource.

[PLT032C7] Eble GJ, Pigliucci M, Preston K (2004). The macroevolution of phenotypic integration. Phenotypic integration: studying the ecology and evolution of complex phenotypes.

[PLT032C38] Fitzgerald MA, Hamilton NRS, Calingacion MN, Verhoeven HA, Butardo VM (2008). Is there a second fragrance gene in rice?. Plant Biotechnology Journal.

[PLT032C8] Fitzgerald MA, McCouch SR, Hall RD (2009). Not just a grain of rice: the quest for quality. Trends in Plant Science.

[PLT032C9] Frankel OH, Soule ME (1981). Conservation and Evolution.

[PLT032C10] Fukuoka S, Suu TD, Ebana K, Trinh LN, Tsukasa N, Kazutoshi O (2006). Diversity in phenotypic profiles in landrace populations of Vietnamese rice: a case study of agronomic characters for conserving crop genetic diversity on farm. Genetic Resources and Crop Evolution.

[PLT032C11] Garris AJ, Tai TH, Coburn J, Kresovich S, McCouch S (2005). Genetic structure and diversity in *Oryza sativa*. Genetics.

[PLT032C12] Hammer Ø, Harper DAT, Ryan PD (2001). PAST, paleontological statistics software package for education and data analysis. Palaeontologia Electronica.

[PLT032C13] Heal G, Walker B, Levin S, Arrow K, Dasgupta P, Daily G, Ehrlich P, Maler K-G, Kautsky N, Lubchenco J, Schneider S, Starrett D (2004). Genetic diversity and interdependent crop choices in agriculture. Resource and Energy Economics.

[PLT032C14] Huang X, Wei X, Sang T, Zhao Q, Feng Qi, Zhao Y, Li C, Zhu C, Lu T, Zhang Z, Li M, Fan D, Guo Y, Wang A, Wang L, Deng L, Li W, Lu Y, Weng Q, Liu K, Huang T, Zhou T, Jing Y, Li Y, Lin Z, Buckler ES, Qian Q, Zhang Q-F, Li J, Han B (2010). Genome-wide association studies of 14 agronomic traits in rice landraces. Nature Genetics.

[PLT032C15] INGER (International Network for Genetic Evaluation of Rice) (1996). Standard evaluation system of rice.

[PLT032C16] IRRI (1980). Descriptors for rice: Oryza sativa L.

[PLT032C17] Jackson MT (1994). Preservation of rice strains. Nature.

[PLT032C18] Jackson MT (1995). Protecting the heritage of rice biodiversity. GeoJournal.

[PLT032C19] Khush GS (1997). Origin, dispersal, cultivation and variation of rice. Plant Molecular Biology.

[PLT032C20] Konishi S, Izawa T, Yang LS, Ebana K, Lin YS, Ebana K, Fukuta Y, Sasaki T, Yano M (2006). An SNP caused loss of seed shattering during rice domestication. Science.

[PLT032C21] Kovach MJ, Sweeney MT, McCouch SR (2007). New insights into the history of rice domestication. Trends in Genetics.

[PLT032C22] Kovach MJ, Calingacion MN, Fitzgerald MA, McCouch SR (2009). The origin and evolution of fragrance in rice (*Oryza sativa* L.). Proceedings of the National Academy of Sciences of the United States of America.

[PLT032C23] Li X, Yan W, Agrama H, Hu B, Jia L, Jia M, Jackson A, Moldenhauer K, McClung A, Wu D (2010). Genotypic and phenotypic characterization of genetic differentiation and diversity in the USDA rice mini-core collection. Genetica.

[PLT032C24] McCouch S (2004). Diversifying selection in plant breeding. PLoS Biology.

[PLT032C25] Meizan K (1985). Genetic structure of African traditional rice cultivars.

[PLT032C26] Neeraja CN, Hariprasad AS, Malathi S, Siddiq EA (2005). Characterization of tall landraces of rice (*Oryza sativa* L.) using gene-derived simple sequence repeats. Current Science.

[PLT032C41] Nguyen VN (2002). Genetic diversity in rice production: case studies from Brazil, India and Nigeria.

[PLT032C27] R Development Core Team (2011). R: A Language and Environment for Statistical Computing.

[PLT032C28] Ray Choudhury P, Kohli S, Srinivasan K, Mohapatra T, Sharma RP (2001). Identification and classification of aromatic rices based on DNA fingerprinting. Euphytica.

[PLT032C40] Reddy CS, Babu AP, Swamy BPM, Kaladhar K, Sarla N (2009). ISSR markers based on GA and AG repeats reveal genetic relationship among rice varieties tolerant to drought, flood, or salinity. Journal of Zhejiang University Science B.

[PLT032C29] Ricklefs RE (2004). Cladogenesis and morphological diversification in passerine birds. Nature.

[PLT032C30] Ricklefs RE (2006). Time, species, and the generation of trait variance in clades. Systematic Biology.

[PLT032C31] Sanni KA, Fawole I, Guei RG, Ojo DK, Somado EA, Tia DD, Ogunbayo SA, Sanchez I (2008). Geographical patterns of phenotypic diversity in *Oryza sativa* landraces of Côte d'Ivoire. Euphytica.

[PLT032C32] Sarla N, Bobba S, Siddiq EA (2003). ISSR and SSR markers based on AG and GA repeats delineate geographically diverse *Oryza nivara* accessions and reveal rare alleles. Current Science.

[PLT032C33] Sharma SD, Tripathy S, Biswal J, Nanda JS (2000). Origin of *O. sativa* and its ecotypes. Rice breeding and genetics: research priorities and challenges.

[PLT032C34] Takano-Kai N, Jiang H, Kubo T, Sweeney M, Matsumoto T, Kanamori H, Padhukasahasram B, Bustamante C, Yoshimura A, Doi K, McCouch S (2009). Evolutionary history of GS3, a gene conferring grain length in rice. Genetics.

[PLT032C35] Yamasaki M, Tenaillon MI, Bi IV, Schroeder SG, Sanchez-Villeda H, Doebley JF, Gaut BS, McMullen MD (2005). A large-scale screen for artificial selection in maize identifies candidate agronomic loci for domestication and crop improvement. The Plant Cell.

[PLT032C36] Zong Y, Chen Z, Innes JB, Chen C, Wang Z, Wang H (2007). Fire and flood management of coastal swamp enabled first rice paddy cultivation in east China. Nature.

