# Supplementary material for: Phenotypic characters of rice landraces reveal independent lineages of short-grain aromatic indica rice
Source: AoB Plants. 2013 Aug 1;5:plt032. doi: 10.1093/aobpla/plt032 (PMC3828656; doi:10.1093/aobpla/plt032)
Supplement: Additional Information [file supp_5_plt032_index.html]

Phenotypic characters of rice landraces reveal independent lineages of short-grain aromatic indica rice — Additional Information 

# Phenotypic characters of rice landraces reveal independent lineages of short-grain aromatic *indica* rice

## Additional Information

**Files in this Data Supplement:**

- Additional Information - Additional Information Table 1
- Additional Information - Additional Information Figure 1
- Additional Information - Additional Information Figure 2
- Additional Information - Additional Information Figure 3
