# Supplementary material for: Phenotypic characters of rice landraces reveal independent lineages of short-grain aromatic indica rice
Source: AoB Plants. 2013 Aug 1;5:plt032. doi: 10.1093/aobpla/plt032 (PMC3828656; doi:10.1093/aobpla/plt032)
Supplement: Additional Information [file supp_plt032_plt032supp_table1.docx]

| **Serial No.** | **Character** | **Abbreviation** | **Unit** |
| --- | --- | --- | --- |
| *Quantitative/ Nominate Descriptors* | | | |
| 1 | Grain length | GL | mm |
| 2 | Grain width | GW | mm |
| 3 | 100-Grain weight | SW | gm |
| 4 | Decorticated grain length | DL | mm |
| 5 | Decorticated grain width | DW | mm |
| 6 | Panicle density | PD | No. Grains/panicle |
| 7 | Awn length | AL | cm |
| 8 | Culm number | CUN | No. Culms/hill |
| 9 | Leaf Length | LL | cm |
| 10 | Plant height | HT | cm |
| 11 | Panicle length | PL | cm |
| 12 | Fraction of sterile (unfilled) grains in panicle | ST% | % |
| 13 | Panicle weight | PW | gm |
| 14 | Leaf length : width ratio | L/W | Number |
| *Qualitative/Ordinate Descriptors* | | | |
| 15 | Lemma-palea pubescence | P | 5 categories |
| 16 | Lemma-palea colour | LP | 9 categories |
| 17 | Apiculus colour | AP | 9 categories |
| 18 | Bran colour | BC | 9 categories |
| 19 | Awn colour | AC | 9 categories |
| 20 | Fragrance | F | 3 categories |
| 21 | Basal leaf sheath colour | BS | 4 categories |
| 22 | Culm internode colour | IC | 3 categories |
| 23 | Leaf angle | LA | 3 categories |
| 24 | Leaf senescence | LS | 3 categories |
| 25 | Flag leaf angle | FA | 3 categories |
| 26 | Culm strength | CS | 3 categories |
| 27 | Secondary branching of panicle | SB | 3 categories |
| 28 | Panicle axis | PX | 4 categories |
| 29 | Panicle threshability | TH | 3 categories |
