# Supplementary figures and images for: Phenotypic characters of rice landraces reveal independent lineages of short-grain aromatic indica rice
Source: AoB Plants. 2013 Aug 1;5:plt032. doi: 10.1093/aobpla/plt032 (PMC3828656; doi:10.1093/aobpla/plt032)

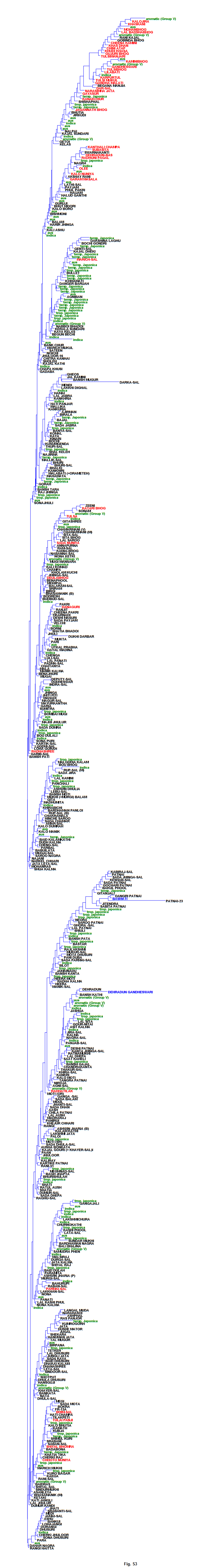

Supplement: Additional Information [file supp_plt032_plt032supp_fig1.tif]

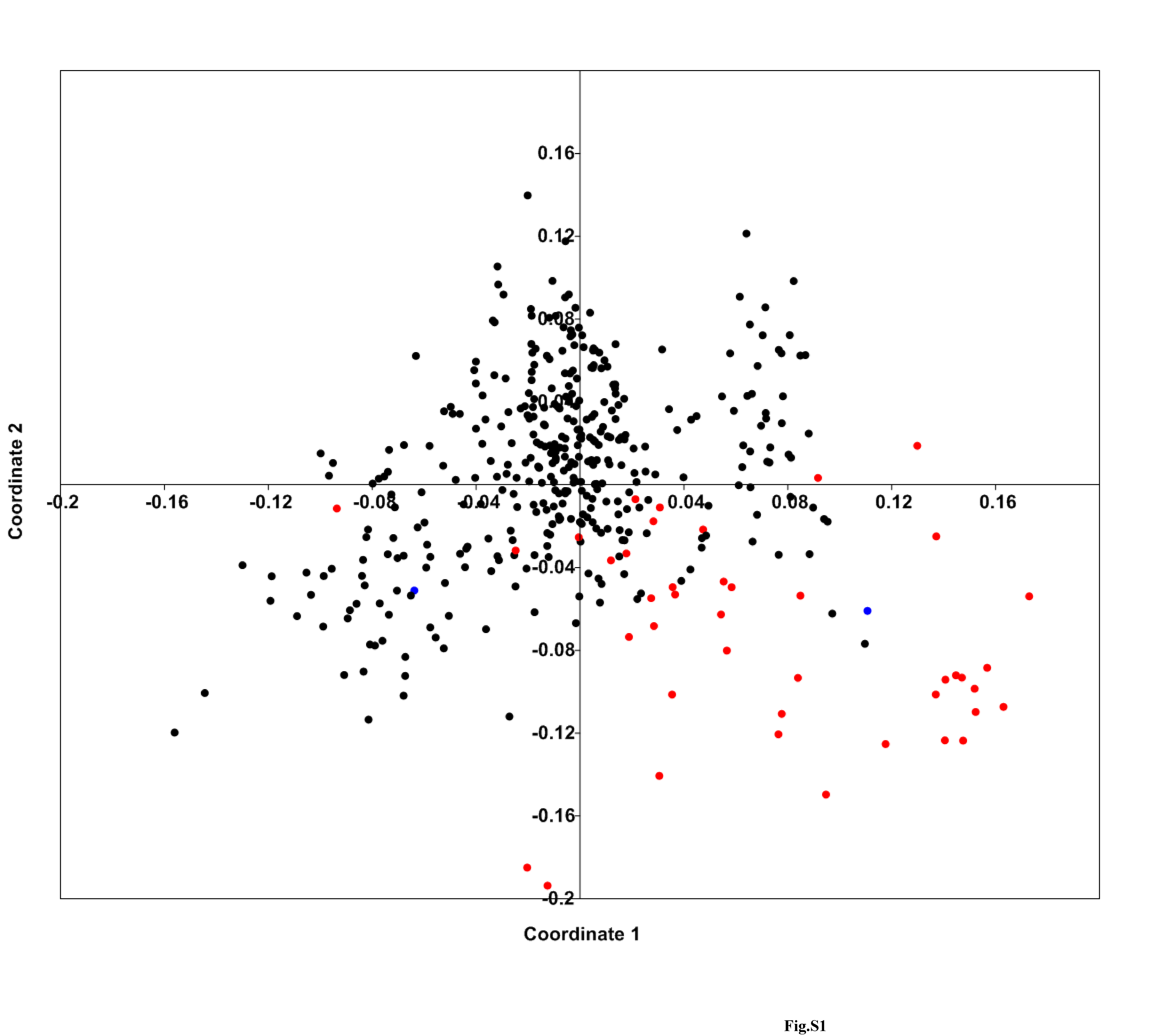

Supplement: Additional Information [file supp_plt032_plt032supp_fig2.tif]
