# Supplementary material for: Phenotypic characters of rice landraces reveal independent lineages of short-grain aromatic indica rice
Source: AoB Plants. 2013 Aug 1;5:plt032. doi: 10.1093/aobpla/plt032 (PMC3828656; doi:10.1093/aobpla/plt032)
Supplement: Additional Information [file supp_plt032_plt032supp_fig3.ppt]

## Slide 1
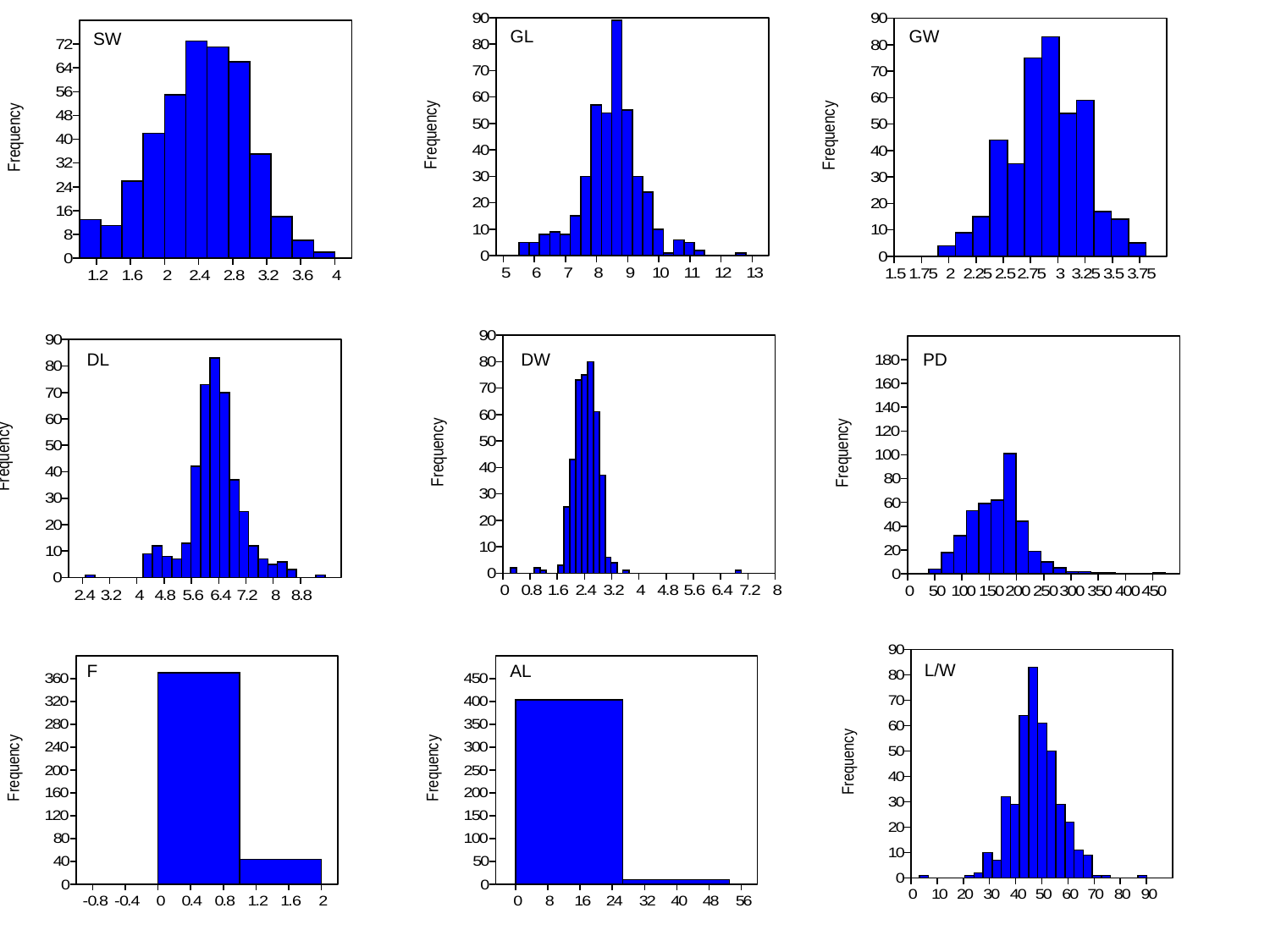

GW
GL
SW
DL
DW
PD
L/W
F
AL

## Slide 2
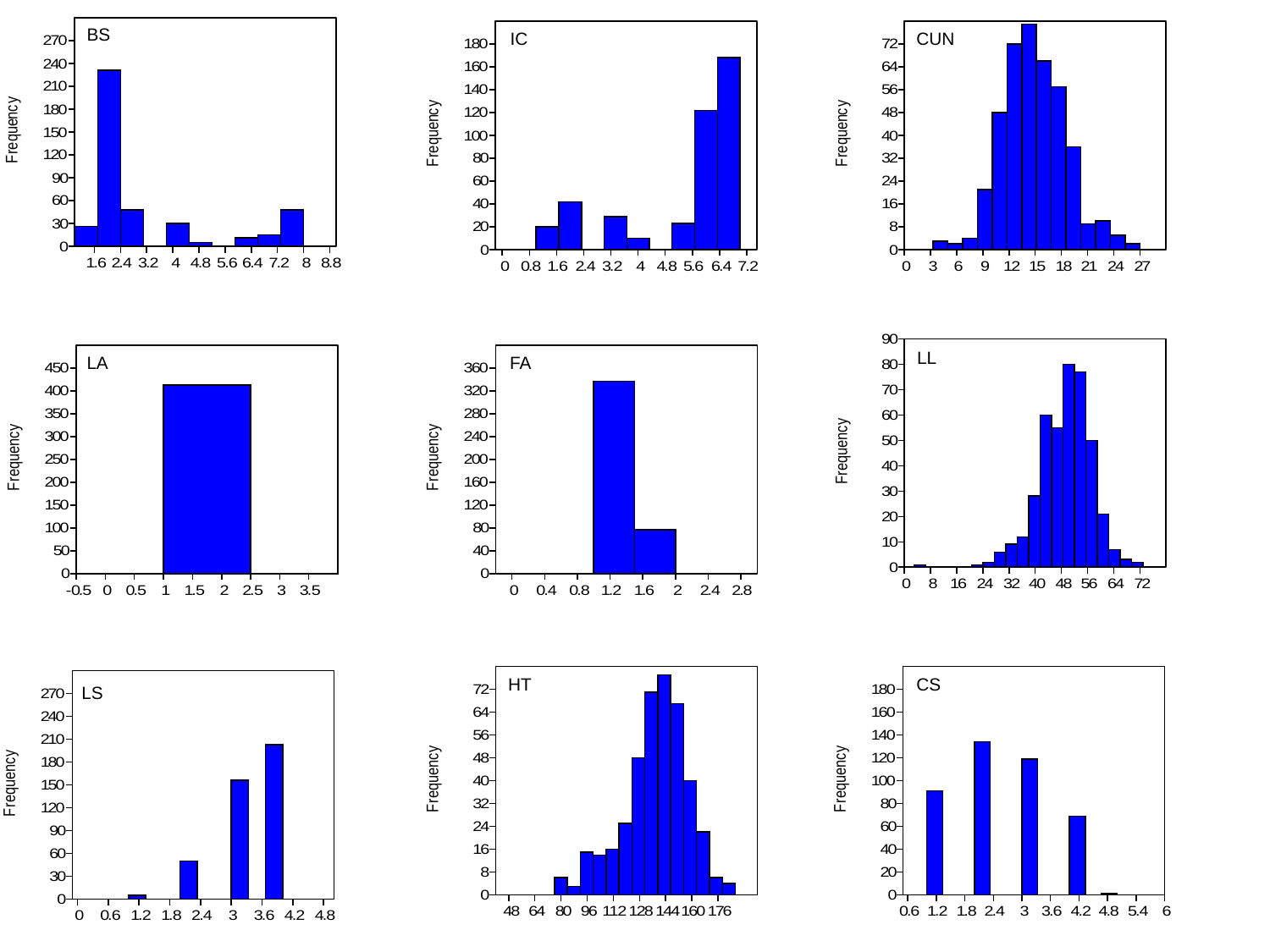

BS
IC
CUN
LL
LA
FA
HT
CS
LS

## Slide 3
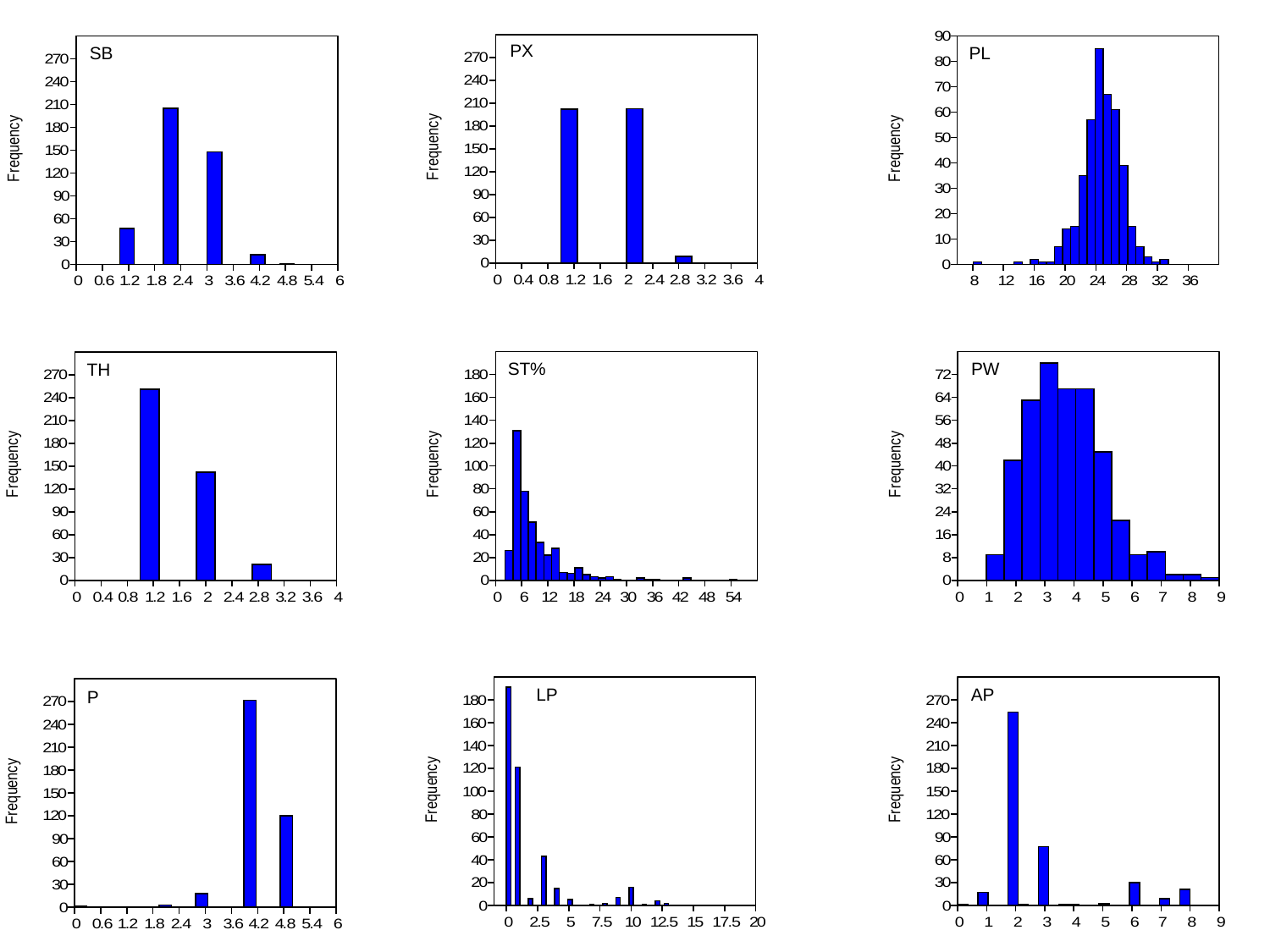

PX
SB
PL
ST%
PW
TH
LP
AP
P

## Slide 4
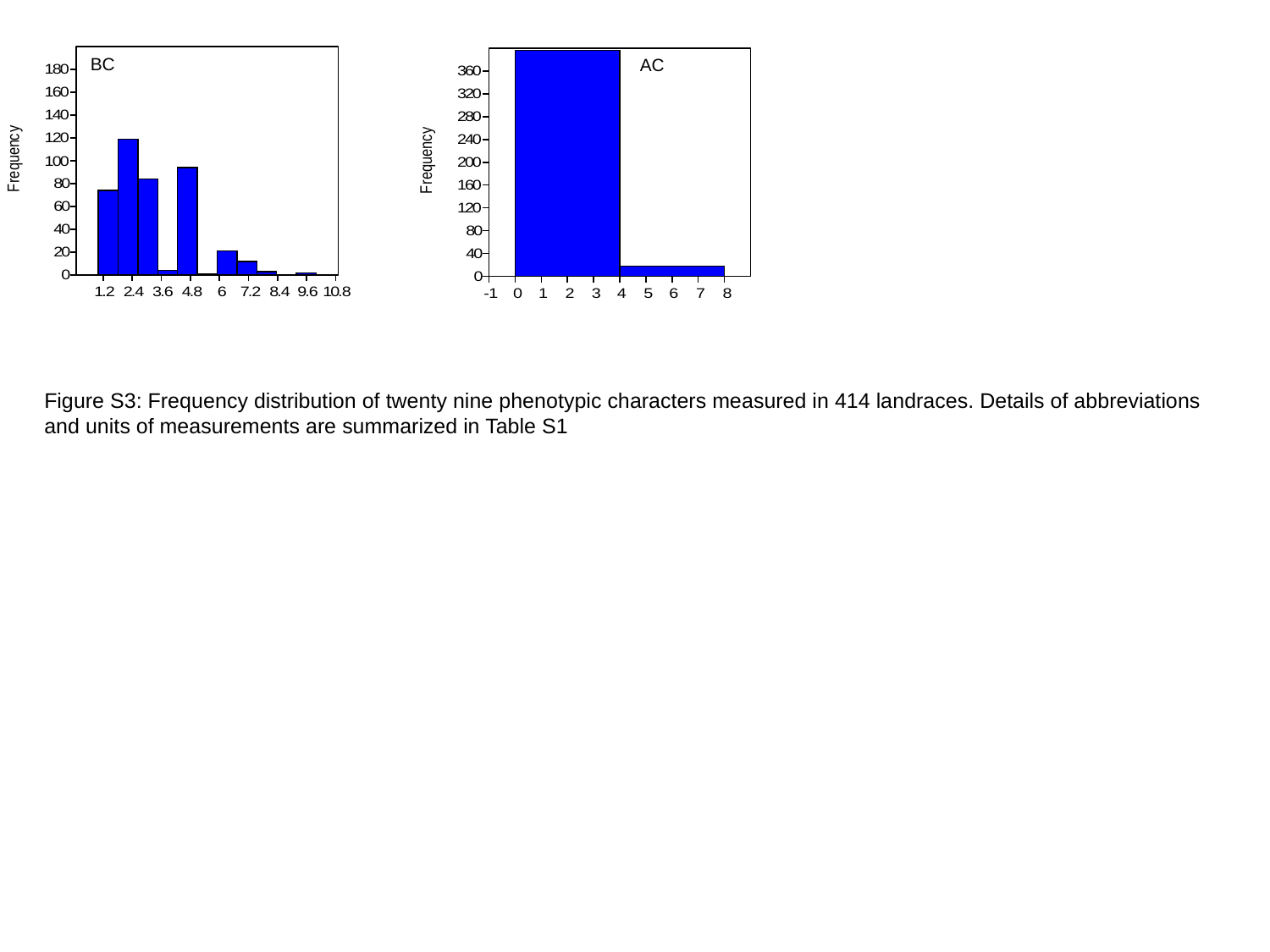

BC
AC
Figure S3: Frequency distribution of twenty nine phenotypic characters measured in 414 landraces. Details of abbreviations and units of measurements are summarized in Table S1
